# Effects of 16 Weeks of Resistance Training on Muscle Quality and Muscle Growth Factors in Older Adult Women with Sarcopenia: A Randomized Controlled Trial

**DOI:** 10.3390/ijerph18136762

**Published:** 2021-06-23

**Authors:** Myong-Won Seo, Sung-Woo Jung, Sung-Woo Kim, Jung-Min Lee, Hyun Chul Jung, Jong-Kook Song

**Affiliations:** 1 Department of Taekwondo, College of Physical Education, Kyung Hee University (Global Campus), 1732 Deokyoungdaero, Giheung-gu, Yongin-si 17104, Korea; myongwonseo@khu.ac.kr; 2 Department of Physical Education, College of Physical Education, Kyung Hee University (Global Campus), 1732 Deokyoungdaero, Giheung-gu, Yongin-si 17104, Korea; jswoo@khu.ac.kr (S.-W.J.); kswrha@khu.ac.kr (S.-W.K.); jungminlee@khu.ac.kr (J.-M.L.); 3 Department of Coaching, College of Physical Education, Kyung Hee University (Global Campus), 1732 Deokyoungdaero, Giheung-gu, Yongin-si 17104, Korea; jhc@khu.ac.kr; 4 Department of Sports & Science, Graduate School of Physical Education, Kyung Hee University (Global Campus), 1732 Deokyoungdaero, Giheung-gu, Yongin-si 17104, Korea

**Keywords:** aging, weight-bearing exercise, elastic bands training, intramuscular fat, follistatin

## Abstract

This study examined the effects of resistance training on muscle quality, muscle growth factors, and functional fitness in older adult women with sarcopenia. Twenty-two older adult women aged over 65 with sarcopenia were randomly assigned to either resistance training (RT, *n* = 12) or non-exercise control group (CG, *n* = 10). The body weight-based and elastic band RT were performed three times a week, 60 min per session, for 16 weeks. Body composition and thigh muscle quality were estimated by dual-energy X-ray absorptiometry (DEXA) and computed tomography (CT), respectively. The muscle growth factors, including growth differentiation factor-8 (GDF-8), growth differentiation factor-15 (GDF-15), activin A, and follistatin, were analyzed via blood samples. Statistical analyses were performed using repeated measures multivariate analysis of variance (MANOVA), analysis of variance (ANOVA), and effect size (i.e., cohen’s *d*, partial eta square), and the significance level was set at 0.05. The RT group improved their functional fitness, grip strength, gait speed, and isometric muscle strength (*p* < 0.01, *d* > 0.99; large), while these variables did not change in the CG. An increase in intramuscular fat was only observed in the CG (*p* < 0.01, 1.06; large). Muscle growth factors such as follistatin were significantly increased in the RT (*p* < 0.05, 0.81; large), but other variables did not change following resistance training. Sixteen weeks of resistance training improved functional fitness and prevented age-related increases in intramuscular fat in the thigh area. However, there were only some changes in muscle growth factors, such as follistatin, suggesting that the effectiveness of resistance training on muscle growth factors is limited. Body weight-based and elastic band resistance training is an alternative training method for sarcopenia to minimize the age-related adverse effects on muscle function and quality.

## 1. Introduction

Resistance training plays an essential role in the potential treatment and prevention strategies of sarcopenia in healthy or sarcopenic older individuals. The assessment of muscle quality and muscle growth factors would add to clinical trials on sarcopenia. Sarcopenia is highly prevalent, and the risk of sarcopenic-related comorbidities is increasing worldwide [1]. The prevalence of sarcopenia is 20% over 70 years old and 30% over 80 years old [2]. Sarcopenia is strongly associated with a progressive decline of skeletal muscle mass and function, resulting in negative consequences such as falls, fractures, morbidity, and mortality [3]. Sedentary lifestyles are well known to increase the risk of developing sarcopenia. Older adult women tend to be more sedentary than older adult men, which is potentially associated with higher mortality risk [4,5]. Additionally, different sex-specific mechanisms such as hormone response and absolute muscle mass influence age-related muscle disease [5,6]. The continuous decline in muscle mass resulted in negative protein balance in the skeletal muscle, but catabolic hormone activity is greater in older women than older men [7]. Therefore, sex-specific treatment strategies for sarcopenia need to be developed to improve quality of life and reduce healthcare costs in older adult women.

Muscle quality is defined as the ability to generate muscle force relative to the volume of contractile tissue [8]. Skeletal muscle quality analysis can identify prognostic parameters and symptoms in sarcopenic older adults compared to healthy individuals. Muscle biopsies provide precise muscle quality information such as fatty infiltration and depletion of muscle and metabolic changes through exercise adaptation. However, they are a burden in human-based research because of their potential risks, including bleeding, bruising, and infection. Therefore, computed tomography (CT) has been used as the gold-standard method for assessing and diagnosing muscle disorder diseases and provide a non-invasive substitute to muscle biopsies [9]. Previous studies suggested that muscle density and intermuscular fat at the quadriceps using CT scans were recommended because they can provide precise clinical diagnosis, evaluation, and degree of disease advancement in sarcopenia [10,11].

The transforming growth factor β (TGF-β) superfamily has been introduced as a primary regulator of muscle architecture involved in extracellular matrix remodeling and cell proliferation regulation [12,13]. GDF-8, GDF-15, and activin A are potent negative regulators of muscle homeostasis in humans [14]. In contrast, follistatin has a decisive role in myostatin-inhibiting activity and muscle hypertrophy [15]. It is believed that the change of these biomarker levels during resistance training could be a novel approach to monitoring geriatric disorders such as sarcopenia, cachexia, and frailty [16,17,18].

Resistance training has been recommended for older individuals to prevent degenerate muscle mass and muscle function [19]. A resistance training program using body weight-based training and/or elastic bands has often been utilized in health care settings. In particular, resistance training using body weight and elastic bands has the potential to be used portably and safely in various populations, and allows participants to perform the exercise anywhere. In addition, it is a secure approach to improve neuromuscular function in the older population [20,21]. Krause et al. demonstrated that 12 weeks of combined body weight-based and elastic band resistance training enhanced lean body mass and muscle function in the older population [20]. Watanabe et al. reported that 16 weeks of body weight-based resistance training improved physical function and muscle strength of the upper and lower extremities in elderly adults [22]. Furthermore, 12 weeks of resistance training using elastic bands improved upper and lower muscle quality (upper; handgrip strength (kg)/arm lean mass (kg), lower; isometric quadriceps (N)/leg lean (kg)) and physical performance in sarcopenic obesity in older women [23]. Thus, we speculated that both body weight-based and elastic band resistance training for sarcopenic older adults could be beneficial to increase muscle function and functional fitness.

Therefore, the present study aimed to determine the effects of body weight-based and elastic band resistance training on muscle quality and muscle growth factors (i.e., GDF-8, GDF-15, activin A, follistatin) in sarcopenic older women. It was hypothesized that 16 weeks of body weight-based and elastic band resistance training enhances functional fitness, muscle function, muscle quality, and muscle growth factors.

## 2. Materials and Methods

### 2.1. Participants

**Figure 1:**
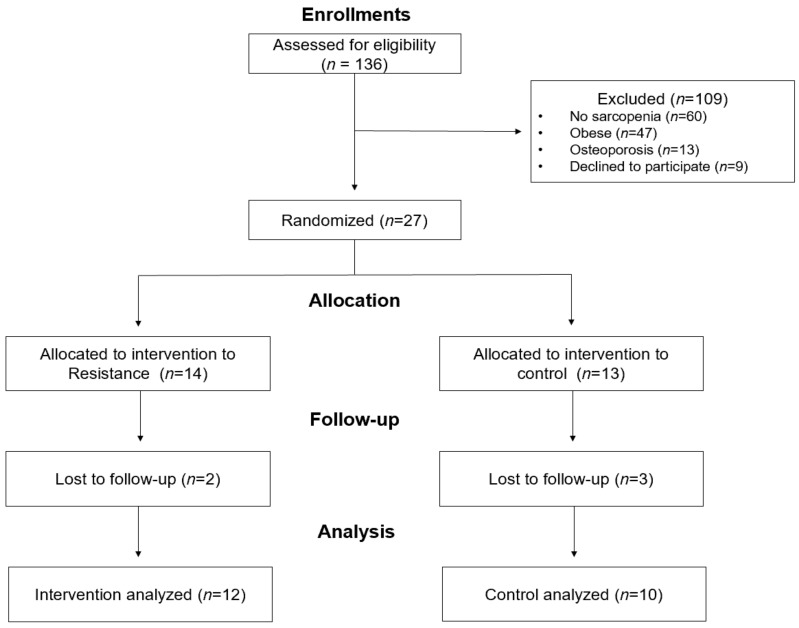
Flow chart of the study.

Participants were recruited through public advertisements at senior citizen centers in South Korea between September 2018 and January 2019. Initially, 136 women aged over 65 enrolled in this study; a pre-enrolment screening questionnaire (AHA/ACSM Health/Fitness Facility Pre-Participation Screening Questionnaire) was used to obtain basic information such as medical history and potential risk factors. Exclusion criteria were as follows: (1) Regular participation in exercise programs at least twice a week (at least 20 min) in the last six months; (2) Either a recent change in the dose of oral hypoglycemic agents, hypertensive drugs, and cholesterol-lowering drugs, or more than 5% weight change within the first two months of the study; (3) Blood pressure over 160/100 mmHg; (4) Communication problems; (5) History of unstable angina pectoris (chest pain), uncontrollable hypertension, type 1 diabetes, heart attack, heart surgery, arthritis, chronic renal failure, epilepsy, convulsions, chronic enterocolitis, and other psychotic disorders associated with loss of mental or cognitive functions. We applied the IWGS (International Working Group on Sarcopenia) and EWGSOP 1 (European Working Group on Sarcopenia in Older People) adjusted diagnostic algorithm to screen eligible participants [10]. The inclusion criteria for sarcopenia diagnosis were (1) gait speed < 1.0 m·s^−1^ and appendicular skeletal muscle index (ASMI) < 5.67 kg·m^−2^, or gait speed > 1.0 m·s^−1^, (2) grip strength < 20 kg and ASMI < 5.67 kg·m^−2^, (3) not obese (percent body fat < 35%), (4) osteoporosis (lumbar or femur bone mineral density T-score < −2.5). After the initial screening process, 100 out of 136 participants did not meet the inclusion criteria. In addition, nine participants refused to participate in the experiments. Thus, twenty-seven participants were randomly assigned to either the resistance training group or the non-exercise control group. The randomization was performed using a computer-generated group assignment. During the experiment, five participants dropped out because of personal reasons or refused post-tests. Consequently, twenty-two sarcopenic older women completed the study (Figure 1). There were no differences in the demographic characteristics between the resistance and non-exercise control groups.

The participant’s characteristics are summarized in Table 1. All participants provided written informed consent before enrollments and were fully informed about the study’s purpose, procedure, benefit, and possible risks by the principal investigator and a research assistant. The Institutional Review Board approved the present study of Kyung Hee University, and informed consent was obtained before screening from all participants.

### 2.2. Anthropometric Measurements

Body height and weight were assessed, with minimal clothing and no shoes, to the nearest 0.1 cm using a stadiometer (T.K.K. Takei Scientific Instruments Co., Tokyo, Japan) and a digital weight scale (150A, CAS, Seoul, South Korea) to the nearest 0.01 kg. Waist circumference (WC) was measured at the midpoint between the iliac crest and the lowest rib margin, and hip circumference (HC) was assessed at the widest point around the buttocks. Based on the anthropometric metric measures, body mass index (BMI; kg·m^−2^) and waist to hip ratio (WHR; waist/hip) were calculated.

### 2.3. Body Composition

Body composition and bone mineral density were estimated by dual-energy X-ray absorptiometry (DXA; QDR-4500W, Hologic, Marlborough, MA, USA). The DXA results included body composition parameters, including fat-free mass, body fat percentage, fat mass, and bone mineral density (whole body, left entire femur, and lumbar spine vertebrae). The appendicular skeletal muscle mass was calculated from the fat-free mass for upper and lower limbs and represented relative to height as ASMI (kg·m^−2^). The coefficient of variance of the DXA machine was 1.5% less as designated by Hologic, Inc. The same experienced technician performed the analysis of the scan, and the intraclass correlation coefficient (ICC) of the DXA estimate was 0.99.

### 2.4. Functional Fitness

The functional fitness was measured using the senior fitness test (SFT), grip strength, and gait speed. The SFT battery tests, including walking (2-min step test), chair stand, chair-sit-and-reach, 2.4 m up and go, and arm curl, were used to measure the participants’ functional fitness [24]. To evaluate sarcopenia for diagnosis purposes, we also assessed 4-m gait speed and grip strength (TKK 5001, Takei, Japan). The ICCs of the functional fitness tests had good to excellent reliability (0.80 to 0.98) [10].

### 2.5. Mid-Thigh Composition

The thigh composition was obtained using 16-slice CT scans (Brivo CT385, GE Healthcare, Chicago, IL, USA) taken from the mid-point of the bilateral thigh between the medial edge of the greater trochanter and intercondyloid. The CT scans estimated the thigh cross-sectional area, total thigh volume (TTV), thigh fat volume (TFV), thigh muscle volume (TMV), thigh subcutaneous fat volume (TSFV), and intramuscular fat (IMAT) in the mid-thigh area. The contradistinction between independent tissue was applied on radiographic attenuation (HU; Hounsfield unit) with a range of −190 to −30 HU for the fat area and a range of 0 to +100 HU for the skeletal muscle area. The CT scans were performed with the radiologist in W hospital, and the same radiologist conducted the analysis.

### 2.6. Maximal Isometric Muscle Strength

The maximal isometric muscle strength was measured using an isokinetic dynamometer (Cybex Humac Norm Model 770, CSMi, Stoughton, MA, USA). Each participant was seated on a dynamometer chair and the torso, forearm, and thigh were tightly fixed with a stabilization pad, with the dominant knee flexed at about 60 degrees. Maximal isometric muscle strength was measured three times for three seconds, and participants had thirty-second static rest between trials. The ICCs were 0.99 for CT scans, and 0.97 for the isometric muscle strength.

### 2.7. Biochemical Markers

All participants were asked to refrain from vigorous physical activity for at least 48 h before collection. Fasting blood samples were obtained at 08:00–09:00 after the participants had 12 h of overnight fasting in our laboratory. Venous blood samples (5 mL) were taken from the antecubital vein into ethylenediaminetetraacetic acid (EDTA) vacuum tubes by the clinical laboratory scientist. The samples were centrifuged at 3000 rpm for 10 min at room temperature, after which plasma samples were immediately separated and stored at −80 °C prior to assay. The plasma blood samples were assessed using an automatic enzyme-linked immunosorbent assay (ELISA) reader (VERSA Max Molecular Devices, Inc., Sunnyvale, CA, USA) with a follistatin kit (Human Follistatin Immunoassay, R&D, Minneapolis, MN, USA), GDF-8 kit (Myostatin Immunoassay ELISA Kit, R&D, Minneapolis, MN, USA), GDF-15 kit (Human GDF-15 Immunoassay, R&D, Minneapolis, MN, USA), and activin A kit (Human Activin A Immunoassay ELISA Kit, R&D, Minneapolis, MN, USA). All biochemical marker assays were conducted at the National Committee Clinic Laboratory (Green Cross Lab Cell, certified by the Korea laboratory accreditation scheme, Yongin-si, South Korea), and the inter- and intraclass coefficient of variance were 5.7% and 1.7% for follistatin, 3.1%, and 1.8% for GDF−8, 4.7% and 1.8% for GDF-15, and 4.7% and 4.2% for activin A.

### 2.8. Intervention

The intervention program was conducted for 16 weeks from March to June in 2019. The resistance training program was performed three times per week over the sixteen-weeks (48 sessions) under the certified strength and conditioning specialist (NSCA-CSCS). Participants were asked to maintain their usual daily activities. Each training session included five minutes of warm-up, fifty minutes of the resistance exercise, and five minutes of cool-down. The weight-bearing exercises described by Watanabe et al. [22] were performed for large muscle groups and further training for small muscle groups was done using an elastic band (Hygenic Corporation, Akron, OH, USA) resistance exercise program [25]. The training load was increased by progressive overload and the OMNI resistance for active muscle scale (OMNI-RES AM, 0-extremely easy to 10-extremely hard) was used [26]. The rest time between sets was 60 s. Details of the exercise program and intensity are shown in Table 2 and Table 3.

### 2.9. Statistical Analysis

All data were analyzed by the SPSS software program (IBM, SPSS version 25, Chicago, IL, USA). Data are expressed as mean, standard deviation (SD), and 95% confidence interval (95% CI). Repeated-measures MANOVA (body composition, functional fitness and physical performance, muscle quality, muscle growth regulator) and ANOVA were applied to determine the interaction effect for the group by the time during intervention periods. A dependent samples t-test was used post-hoc if significant interaction effects were detected. The effect sizes were expressed as partial eta-squared values within repeated measures ANOVA squared (η^2^_p_; small ≥ 0.01, medium ≥ 0.06, large ≥ 0.14) and Cohen’s *d* (*d*; small ≥ 0.2, medium ≥ 0.5, large ≥ 0.8) was used to indicate the mean difference between groups. A statistically significant level was defined as 0.05.

## 3. Results

Repeated measures MANOVA demonstrated that there was no significant interaction effects on anthropometric parameters, body composition, and bone mineral density. There were also no significant interaction effects of group and time on the BMC and BMD of three regions (whole-body, lumbar, and femur). However, there was a significant interaction effect for the group by time on WHR (F(1,20) = 7.188, *p* = 0.014, η^2^_p_ = 0.264). The CG increased WHR (*p* < 0.01, *d* = 1.04) after 16 weeks compared with the RT (Table 4).

Two-way repeated measures MANOVA revealed significant interaction effects on functional fitness and physical performance (F(1,20) = 10.880, *p* < 0.001, η^2^_p_ = 0.813) (Table 5). Seven univariate variables were found to have significant interaction effects for group by time, including 30-s chair stand (F(1,20) = 20.608, *p* < 0.001, η^2^_p_ = 0.507), 30-s arm curl (F(1,20) = 45.996, *p* < 0.001, η^2^_p_ = 0.697), chair sit-and-reach (F(1,20) = 37.101, *p* < 0.001, η^2^_p_ = 0.650), 8-foot up-and-go (F(1,20) = 29.831, *p* < 0.001, η^2^_p_ = 0.599), 2-min step test (F(1,20) = 22.453, *p* < 0.001, η^2^_p_ = 0.529), grip strength (F(1,20) = 41.123, *p* < 0.001, η^2^_p_ = 0.673), and gait speed (F(1,20) = 23.635, *p* < 0.001, η^2^_p_ = 0.542). As mentioned above, all variables improved significantly in the RT group (*p* < 0.001, *d* > 1.20), and chair sit-and-reach, 2.4-m up-and-go, 2-min step, and grip strength were degenerated in the CG (*p* < 0.05, *d* > 0.79).

Repeated measures MANOVA showed significant interaction effects on mid-thigh muscle quality (F(1,20) = 7.579, *p* < 0.05, η^2^_p_ = 0.752)(Table 6). There were significant interaction effects for group by time in maximum voluntary isometric contraction (MVIC) (F(1,20) = 7.417, *p* = 0.013, η^2^_p_ = 0.271) and relative maximum voluntary isometric contraction (RMVIC) (F(1,20) = 7.560, *p* = 0.012, η^2^_p_ = 0.274). RT improved MVIC (*p* < 0.01, Cohen’s *d* = 1.09) and RMVIC (*p* < 0.01, Cohen’s *d* = 1.20) during the intervention period compared with CG. Furthermore, significant interaction effects for group by time were shown for TMV (F(1,20) = 4.872, *p* = 0.039, η^2^_p_ = 0.196) and IMAT (F(1,20) = 7.381, *p* = 0.013, η^2^_p_ = 0.270) during the intervention. However, TMV did not change in both groups, and IMAT increased significantly in the CG (*p* < 0.01, *d* = 1.06) after 16 weeks.

A repeated measures MANOVA indicated no significant interaction effects for the group by time for GDF-8, GDF-15, and activin A, and no significant interaction effects for muscle growth regulators (Table 6). However, there was a significant interaction effect for follistatin (F(1,20) = 4.960, *p* = 0.038, η^2^_p_ = 0.199) during the intervention period. Follistatin improved significantly in the RT (*p* < 0.05, Cohen’s *d* = 0.81) compared with the CG during the intervention.

## 4. Discussion

A main finding of the present study is that 16 weeks of resistance training using body weight-based training and elastic bands significantly improves muscle quality and functional fitness in sarcopenic older women. However, it remains unclear whether our training protocol affects muscle growth factors. Therefore, we accept our hypotheses that a change in functional fitness and muscle quality in sarcopenic older adults would be seen after 16 weeks of resistance training compared with the control group. Based on the results of this study, we reject the hypothesis that 16 weeks of resistance training affected muscle growth factors in sarcopenic older women.

Resistance training has been recommended for older adults, but several studies have failed to show an increase in skeletal muscle mass, particularly in sarcopenic older adults [22,27]. However, Vikberg et al. demonstrated that pre-sarcopenic older adults improved their fat-free mass, fat mass, and ASMI compared with the control group following ten weeks of resistance training [21]. It has been inconsistently observed that participants’ characteristics, intervention periods, and nutritional intake may influence the skeletal muscle mass. The increase in skeletal muscle mass could have been affected by the nutrition intake supplied to the individual during the intervention period and the various exercise programs used by older adults [28]. A previous study observed that 12 weeks of strength exercises with nutritional supplementation (i.e., whey protein, essential amino acids, vitamin D) improved fat-free mass compared with the placebo group in older adults [29], and combined interventions with exercise and dietary intake have been shown to induce a positive change in the muscle mass of older adults with sarcopenia [30,31]. In addition, no significant interaction effects of bone mineral density and bone mineral content were identified in sarcopenic older adults, representing no bone parameter changes. Similar results have been observed by Beavers et al. and Strandberg et al. [32,33]. The authors speculated that bone composition changes could be expected from long-term intervention periods of at least 24 weeks, and efficient bone remodeling after 6 to 8 months leads to new steady-state bone after intervention periods. Although the present study did not show the interaction effects of bone composition, we assume that 16 weeks of non-exercising in thecontrol group had significant adverse effects during the intervention (lumbar BMC; *p* < 0.01, Cohen’s *d* =1.09). The importance of functional fitness has been accentuated as a major factor in lifestyle diseases and functional mobility [24]. The present study revealed that resistance training using body weight-based training and elastic bands improves functional fitness and physical performance in sarcopenic older women (F(1,20) = 10.880, *p* < 0.001, η^2^_p_ = 0.813). Resistance training is a potential treatment with immense evidence of improving functional mobility in older adults. Previous studies indicated that 12 weeks of resistance training significantly increased grip strength, power, agility, flexibility, and functional performance in older adults [34]. However, Martins et al. found that eight weeks of resistance training using elastic bands did not change grip strength [35], and Vikberg et al. also showed that there were no significant changes in the short physical performance battery (SPPB) score after the resistance training over 10 weeks in pre-sarcopenic older adults [21]. The conflicting results may derive from the different types of exercises, intensities, frequencies, intervention periods, and participants’ pathological conditions. Nevertheless, our study confirmed that 16 weeks of body weight-based and elastic band resistance training had a positive effect on the independence of daily living by the improvement of functional fitness.

Muscle quality, generally defined as muscle strength per unit of muscle mass, is the substantial determinant of muscle function, and it decreases with aging and affects quality of life in older adults with sarcopenia [36]. Notably, we observed that the muscle function significantly improved with a 16-week intervention period (F(1,20) = 4.771, *p* < 0.01, η^2^_p_ = 0.656). Although univariate variable analysis indicated no significant change between baseline and post-tests on TTV, TFV, and TSFV, the isometric muscle strength was largely improved in the resistance training group compared with the control group. Moreover, resistance training using body weight-based training and elastic bands may maintain the age-related increases in IMAT levels, which were shown to increase in the control group (Cohen’s *d* = 1.06). Previous studies demonstrated that IMAT had been associated with lower muscle strength, mobility function, and various diseases (i.e., heart failure, diabetes, and cancer) [37,38]. As a result, muscle function improves after resistance training, which can positively affect muscle architecture. We assume that maintaining IMAT levels is essential to consider because they may be partially accountable for muscle function with resistance exercise and have potential clinical implications and prognostic value in various pathologies. This study confirmed that 16 weeks of suggested resistance training should mitigate the accretion of IMAT in sarcopenic older adults.

Although MANOVA for the group by time for muscle growth factors revealed no interaction effects, the univariate variable analysis indicated a significant group by time interaction on follistatin. The RT group greatly increased follistatin following 16 weeks of intervention (Cohen’s *d* = 0.81), whereas this parameter did not change in the CG. Similar to the present study, Hofmann et al. observed a significant increase in follistatin, whereas GDF-8, GDF-15, and activin A did not change after six months of intervention in resistance training with older adult women [39]. Physiological processes are activated after exercise, including energy homeostasis and restoring skeletal muscle remodeling; therefore, an increase in follistatin expression after the intervention is positive for muscle growth factors. We also observed that the control group showed a large increase in GDF-15 in this study (Cohen’s *d* = 0.83); however, this requires careful interpretation because there was no significant group by time interaction. The hormone response of follistatin, GDF-8, GDF-15, and activin A to resistance training may depend on the training protocol, exercise load, duration, periods, participants’ characteristics (e.g., physical activity, gender, and muscle mass), and health status during the training and recovery periods. Higher TGF-β superfamily member levels were associated with the existence of various diseases [40]. Regarding muscle growth regulators, it is unclear why 16 weeks of body weight-based and elastic band resistance training only increases follistatin but not all TGF-β superfamily members; however, the results indicate that the training protocol was sufficient to improve muscle strength. Thus, in the future, proper high-intensity long-term exercise interventions should be applied to examine the changes in muscle growth factors and muscle hypertrophy.

Notably, a strength of the present study is that all participants in the resistance group showed improved muscle quality. However, the potential limitation of the present study is that the sample size is small with sarcopenic older adult women and physical activities and dietary intake not tightly controlled. In addition, we recruited 136 women over 65 years for the initial screening. However, using the Asian Working Group for Sarcopenia (AWGS, 5.9%), EWGSOP (7.4%), and Foundation for the National Institutes of Health (FNIH, 4.4%) diagnosis, very few participants were classified as sarcopenic in the present study. Thus, this study chose to use an adjusted diagnostic algorithm based on IWGS and EWGSOP, which may have limited the present study’s power to detect a difference between groups.

## 5. Conclusions

In conclusion, 16 weeks of body weight-based and elastic band resistance training improved functional fitness and muscle quality in older women with sarcopenia. However, there were only some changes in muscle growth factors, such as follistatin, suggesting that the effectiveness of resistance training on muscle growth factors is limited. Resistance training with body weight-based training and elastic bands can be an alternative and practical method for sarcopenia prevention, minimizing the age-related adverse effects on muscle function and quality.

## Figures and Tables

**Table 1:** Baseline characteristics of the participants (M ± SD).

Variables	RT (*n* = 12)	CON (*n* = 10)	*p*-Value
Age (yrs)	70.3 ± 5.38	72.9 ± 4.75	0.239
Body height (cm)	152.9 ± 4.92	151.4 ± 5.45	0.505
Body weight (kg)	53.4 ± 4.39	51.5 ± 5.64	0.396
BMI (kg·m^−2^)	22.9 ± 2.02	22.4 ± 1.52	0.579
Waist circumference (cm)	76.2 ± 5.18	77.4 ± 4.56	0.564
Hip circumference (cm)	89.6 ± 4.38	90.6 ± 4.27	0.598
WHR	0.85 ± 0.04	0.85 ± 0.03	0.804

Note: RT: resistance training group, CON: control group, BMI; body mass index, WHR; waist-hip ratio.

**Table 2:** Bodyweight-based and elastic resistance training program.

Exercise Program	Type	Part	Contents
	(Time)		
Warm-Up	Stretching (5 min)	Whole-Body	Stretching, Walking
Main exercise	Resistance (50 min)	Upper body	Shoulder press, Front raise, Lateral raise, Biceps curl, Triceps extension, Kick back, Crunch, Bent over row, Seated row, Back extension (prone), Push up (beginner)
		Lower body	Squat, Lunge, Lying leg abduction, Leg kick back, Pelvic lift, Leg raise, Toe & Heel raise
Cool-down	Stretching (5 min)	Whole-body	Static stretching

**Table 3:** Training protocol.

Exercise	Phase (weeks)	Repetitions	Sets	Intensity
				(OMNI Scale/Color)
Resistance training	1	6	3	4/Yellow
	2	8	3	4/Yellow
	3	10	3	5/Yellow
	4	12	3	5/Yellow
	5	10	4	6/Yellow
	6	10	4	6/Yellow
	7	12	5	6/Yellow
	8	12	5	6/Yellow
	9	15	3	7/Yellow
	10	15	3	7/Yellow
	11	15	3	7/Yellow
	12	15	3	7/Yellow
	13	15	4	8/Yellow
	14	15	4	8/Yellow
	15	15	5	8/Yellow
	16	15	5	8/Yellow

**Table 4:** Change in participant characteristics, body composition, and bone mineral density during the intervention (M ± SD).

Variables	Group	Baseline	Post	*d*	Univariate Interaction (η^2^_p_)	Manova Interaction (η^2^_p_)
Body weight (kg)	RT	53.4 ± 4.39	53.3 ± 4.39	0.07	0.072 (0.004)	1.392 (0.247)
	CG	51.5 ± 5.64	51.3 ± 5.95	0.15		
BMI (kg·m^−2^)	RT	22.9 ± 2.02	22.9 ± 2.02	0.01	0.019 (0.001)	
	CG	22.4 ± 1.52	22.4 ± 1.58	0.04		
Waist circumference (cm)	RT	76.2 ± 5.18	76.1 ± 4.18	0.04	2.068 (0.094)	
	CG	77.4 ± 4.56	78.4 ± 5.60	0.71		
Hip circumference (cm)	RT	89.6 ± 4.38	89.9 ± 3.68	0.17	1.593 (0.074)	
	CG	90.6 ± 4.27	90.1 ± 4.00	0.42		
WHR	RT	0.85 ± 0.04	0.85 ± 0.04	0.19	7.188 * (0.264)	
	CG	0.85 ± 0.03	0.87 ± 0.04 ^++^	1.04		
Fat mass (kg)	RT	19.1 ± 3.11	19.2 ± 3.21	0.12	0.288 (0.014)	0.397 (0.062)
	CG	17.5 ± 3.58	17.4 ± 3.77	0.11		
Fat-free mass (kg)	RT	31.6 ± 2.10	31.9 ± 1.87	0.46	1.414 (0.066)	
	CG	31.5 ± 2.58	31.5 ± 2.87	0.10		
ASM (kg)	RT	12.3 ± 0.96	12.4 ± 0.77	0.25	2.645 (0.117)	
	CG	12.4 ± 0.95	12.2 ± 1.10	0.43		
Percent body fat (%)	RT	36.3 ± 3.74	36.3 ± 3.82	0.02	0.028 (0.001)	
	CG	34.4 ± 3.85	34.3 ± 4.14	0.08		
Whole-body BMC (g)	RT	1574.36 ± 193.79	1566.75 ± 189.47	0.35	0.421 (0.021)	1.083 (0.253)
	CG	1460.46 ± 210.27	1446.86 ± 206.42	0.63		
Whole body BMD (g·cm^−2^)	RT	0.951 ± 0.071	0.949 ± 0.071	0.11	0.108 (0.005)	
	CG	0.915 ± 0.058	0.916 ± 0.061	0.03		
Lumbar BMC (g)	RT	47.71 ± 9.15	47.48 ± 9.47	0.14	1.965 (0.089)	
	CG	41.50 ± 8.46	40.46 ± 7.88	0.09		
Lumbar BMD (g·cm^−2^)	RT	0.834 ± 0.110	0.833 ± 0.121	0.04	0.589 (0.029)	
	CG	0.761 ± 0.102	0.755 ± 0.097	0.40		
Femur BMC (g)	RT	23.01 ± 3.53	22.97 ± 3.03	0.03	0.031 (0.002)	
	CG	20.09 ± 3.73	19.98 ± 3.53	0.15		
Femur BMD (g·cm^−2^)	RT	0.729 ± 0.090	0.734 ± 0.085	0.26	0.168 (0.008)	
	CG	0.650 ± 0.105	0.652 ± 0.104	0.31		

Values are expressed as mean ± SD, * Significant interaction effect, * *p* < 0.05, ^+^ Significant difference between pre- and post-test, ^++^ *p* < 0.01, BMI; body mass index, WHR; waist hip ratios, ASM; appendicular skeletal muscle index, BMC; bone mineral contents, BMD; bone mineral density, Cohen’s *d*; small ≥ 0.2, medium ≥ 0.5, large ≥ 0.8, η^2^_p_; small ≥ 0.01, medium ≥ 0.06, large ≥ 0.14.

**Table 5:** Changes in functional fitness and physical performance during the intervention.

Variables	Group	Baseline	Post	*d*	Univariate Interaction (η^2^_p_)	Manova Interaction (η^2^_p_)
30-s chair stand (*n*)	RT	15.5 ± 4.50	20.3 ± 5.16 ^+++^	1.69	20.608 *** (0.507)	10.880 *** (0.813)
	CG	12.1 ± 3.07	12.1 ± 3.14	0.00		
30-s arm curl (*n*)	RT	16.3 ± 3.68	21.2 ± 3.69 ^+++^	2.48	45.996 *** (0.697)	
	CG	13.9 ± 3.25	13.1 ± 2.60	0.41		
Chair sit-and-reach (cm)	RT	19.8 ± 7.15	24.3 ± 6.94 ^++^	1.39	37.101 *** (0.650)	
	CG	6.2 ± 10.56	3.5 ± 10.08 ^++^	1.33		
8-foot up-and-go (s)	RT	5.7 ± 0.45	5.1 ± 0.45 ^+++^	1.77	29.831 *** (0.599)	
	CG	5.9 ± 0.65	6.3 ± 0.77 ^+^	0.79		
2-min step test (*n*)	RT	91.3 ± 11.01	108.3 ± 11.04 ^++^	1.20	22.453 *** (0.529)	
	CG	86.7 ± 12.96	75.1 ± 18.92 ^+^	0.83		
Grip strength (kg)	RT	20.8 ± 2.93	24.3 ± 2.25 ^+++^	1.72	41.123 *** (0.673)	
	CG	18.6 ± 3.07	17.3 ± 3.61 ^+^	0.99		
Gait speed (m·s^−1^)	RT	0.96 ± 0.08	1.14 ± 0.11 ^+++^	1.84	23.635 *** (0.542)	
	CG	0.93 ± 0.09	0.95 ± 0.09	0.43		

Values are expressed as mean ± SD, * Significant interaction effect, *** *p* < 0.001, ^+^ Significant difference between pre- and post-test, ^+^ *p* < 0.05, ^++^ *p* < 0.01, ^+++^ *p* < 0.001, Cohen’s *d*; small ≥ 0.2, medium ≥ 0.5, large ≥ 0.8, η^2^_p_; small ≥ 0.01, medium ≥ 0.06, large ≥ 0.14.

**Table 6:** Change in muscle quality and muscle growth regulators during the intervention.

Variables	Variables	Group	Baseline	Post	*d*	Univariate Interaction (η^2^_p_)	Manova Interaction (η^2^_p_)
Mid-Thigh Muscle Quality	MVIC (N·m)	RT	112.4 ± 21.60	123.5 ± 18.87 ^+^	1.09	7.417 (0.271)	
		CG	97.6 ± 30.94	93.7 ± 25.47	0.25		
	RMVIC (N·m·kg^−1^)	RT	209.9 ± 32.18	231.8 ± 30.25 ^+^	1.20	7.560 (0.274)	
		CG	195.4 ± 76.53	186.6 ± 61.29	0.26		
	TTV (cm^2^)	RT	142.3 ± 17.60	142.5 ± 19.28	0.02	0.168 (0.008)	
		CG	138.5 ± 20.12	140.2 ± 15.56	0.25		
	TFV (cm^2^)	RT	62.9 ± 15.43	61.7 ± 15.97	0.19	2.965 (0.129)	7.579 * (0.752)
		CG	60.7 ± 13.00	64.5 ± 9.52	0.53		
	TMV (cm^2^)	RT	74.8 ± 6.77	76.6 ± 7.48	0.44	4.872 * (0.196)	
		CG	72.3 ± 8.86	71.0 ± 9.48	0.71		
	TSFV (cm^2^)	RT	46.9 ± 17.36	46.3 ± 16.09	0.07	0.585 (0.028)	
		CG	44.7 ± 10.89	46.4 ± 7.41	0.27		
	IMAT (cm^2^)	RT	16.0 ± 4.60	15.4 ± 3.15	0.25	7.381 * (0.270)	
		CG	16.1 ± 3.90	18.1 ± 3.75 ^++^	1.06		
Muscle Growth Regulator	Follistatin (pg·mL^−1^)	RT	2113.75 ± 409.28	2652.85 ± 704.18 ^+^	0.81	4.960 * (0.199)	2.476 (0.292)
		CG	2241.85 ± 669.91	2255.45 ± 564.02	0.04		
	GDF-8 (pg·mL^−1^)	RT	2294.43 ± 686.62	2193.11 ± 618.91	0.19	1.494 (0.070)	
		CG	1616.52 ± 650.70	1784.22 ± 529.69	0.33		
	GDF-15 (pg·mL^−1^)	RT	902.00 ± 406.93	961.81 ± 355.81	0.43	0.943 (0.045)	
		CG	831.03 ± 262.84	949.32 ± 326.74	0.83		
	Activin A (pg·mL^−1^)	RT	399.81 ± 93.59	362.42 ± 76.83	0.45	0.011 (0.001)	
		CG	340.37 ± 61.61	305.94 ± 53.01	0.90		

Values are expressed as mean ± SD, MVIC; maximum voluntary isomeric contraction, RMVIC; relative maximum voluntary isomeric contraction, TTV; thigh cross-sectional area total thigh volume, TFV; thigh fat volume TMV; thigh muscle volume, TSFV; thigh subcutaneous fat volume, IMAT; intramuscular fat, * Significant interaction effect, * *p* < 0.05, ^+^ Significant difference between pre- and post-test, ^+^ *p* < 0.05, ^++^ *p* < 0.01, Cohen’s *d*; small ≥ 0.2, medium ≥ 0.5, large ≥ 0.8, η^2^_p_; small ≥ 0.01, medium ≥ 0.06, large ≥ 0.14.

## Data Availability

The raw data will be available at reasonable request from the corresponding author.

## References

[1] Shafiee G., Keshtkar A., Soltani A., Ahadi Z., Larijani B., Heshmat R. (2017). Prevalence of sarcopenia in the world: A systematic review and meta- analysis of general population studies. J. Diabetes Metab. Disord..

[2] Dodds R.M., Roberts H.C., Cooper C., Sayer A.A. (2015). The Epidemiology of Sarcopenia. J. Clin. Densitom..

[3] Yeung S.S., Reijnierse E.M., Pham V.K., Trappenburg M.C., Lim W.K., Meskers C.G., Maier A.B. (2019). Sarcopenia and its association with falls and fractures in older adults: A systematic review and meta-analysis. J. Cachexi Sarcopenia Muscle.

[4] Lee Y.S. (2005). Gender differences in physical activity and walking among older adults. J. Women Aging.

[5] Batsis J.A., Mackenzie T.A., Barre L.K., Lopez-Jimenez F., Bartels S.J. (2014). Sarcopenia, sarcopenic obesity and mortality in older adults: Results from the National Health and Nutrition Examination Survey III. Eur. J. Clin. Nutr..

[6] Drescher C., Konishi M., Ebner N., Springer J. (2015). Loss of muscle mass: Current developments in cachexia and sarcopenia focused on biomarkers and treatment. J. Cachexi Sarcopenia Muscle.

[7] Payette H., Roubenoff R., Jacques P.F., Dinarello C.A., Wilson P.W., Abad L.W., Harris T. (2003). Insulin-like growth factor-1 and interleukin 6 predict sarcopenia in very old community-living men and women: The Framingham Heart Study. J. Am. Geriatr. Soc..

[8] Hill C., James R.S., Cox V.M., Seebacher F., Tallis J. (2020). Age-related changes in isolated mouse skeletal muscle function are dependent on sex, muscle, and contractility mode. Am. J. Physiol. Regul. Integr. Comp. Physiol..

[9] Heymsfield S.B., Adamek M., Gonzalez M.C., Jia G., Thomas D.M. (2014). Assessing skeletal muscle mass: Historical overview and state of the art. J. Cachexi Sarcopenia Muscle.

[10] Seo M.W., Jung S.W., Kim S.W. (2020). Comparisons of Muscle Quality and Muscle Growth Factor between Sarcopenic and Non-Sarcopenic Older Women. Int. J. Environ. Res. Public Health.

[11] Albano D., Messina C., Vitale J., Sconfienza L.M. (2020). Imaging of sarcopenia: Old evidence and new insights. Eur. Radiol..

[12] Parker L., Caldow M.K., Watts R., Levinger P., Cameron-Smith D., Levinger I. (2017). Age and sex differences in human skeletal muscle fibrosis markers and transforming growth factor-β signaling. Eur. J. Appl. Physiol..

[13] Ismaeel A., Kim J.-S., Kirk J.S., Smith R.S., Bohannon W.T., Koutakis P. (2019). Role of Transforming Growth Factor-β in Skeletal Muscle Fibrosis: A Review. Int. J. Mol. Sci..

[14] Latres E., Mastaitis J. (2017). Activin A more prominently regulates muscle mass in primates than does GDF8. Nat. Commun..

[15] Han X., Møller L.L.V. (2019). Mechanisms involved in follistatin-induced hypertrophy and increased insulin action in skeletal muscle. J. Cachexia Sarcopenia Muscle.

[16] Calvani R., Marini F., Cesari M., Tosato M., Anker S.D., von Haehling S., Miller R.R., Bernabei R., Landi F., Marzetti E. (2015). Biomarkers for physical frailty and sarcopenia: State of the science and future developments. J. Cachexi Sarcopenia Muscle.

[17] Ebner N., Steinbeck L., Doehner W., Anker S.D., von Haehling S. (2014). Highlights from the 7th Cachexia Conference: Muscle wasting pathophysiological detection and novel treatment strategies. J. Cachexi Sarcopenia Muscle.

[18] Mafi F., Biglari S., Ghardashi Afousi A., Gaeini A.A. (2019). Improvement in Skeletal Muscle Strength and Plasma Levels of Follistatin and Myostatin Induced by an 8-Week Resistance Training and Epicatechin Supplementation in Sarcopenic Older Adults. J. Aging Phys. Act..

[19] Hughes L., Paton B., Rosenblatt B., Gissane C., Patterson S.D. (2017). Blood flow restriction training in clinical musculoskeletal rehabilitation: A systematic review and meta-analysis. Br. J. Sports Med..

[20] Krause M., Crognale D., Cogan K., Contarelli S., Egan B., Newsholme P., De Vito G. (2019). The effects of a combined bodyweight-based and elastic bands resistance training, with or without protein supplementation, on muscle mass, signaling and heat shock response in healthy older people. Exp. Gerontol..

[21] Vikberg S., Sörlén N., Brandén L., Johansson J., Nordström A., Hult A., Nordström P. (2019). Effects of Resistance Training on Functional Strength and Muscle Mass in 70-Year-Old Individuals With Pre-sarcopenia: A Randomized Controlled Trial. J. Am. Med. Dir. Assoc..

[22] Watanabe Y., Tanimoto M., Oba N., Sanada K., Miyachi M., Ishii N. (2015). Effect of resistance training using bodyweight in the elderly: Comparison of resistance exercise movement between slow and normal speed movement. Geriatr. Gerontol. Int..

[23] Liao C.D., Tsauo J.Y., Huang S.W., Ku J.W., Hsiao D.J., Liou T.H. (2018). Effects of elastic band exercise on lean mass and physical capacity in older women with sarcopenic obesity: A randomized controlled trial. Sci. Rep..

[24] Rikli R.E., Jones C.J. (2013). Development and validation of criterion-referenced clinically relevant fitness standards for maintaining physical independence in later years. Gerontologist.

[25] Park Y.H., Song M., Cho B.L., Lim J.Y., Song W., Kim S.H. (2011). The effects of an integrated health education and exercise program in community-dwelling older adults with hypertension: A randomized controlled trial. Patient Educ. Couns..

[26] Colado J.C., Garcia-Masso X., Triplett T.N., Flandez J., Borreani S., Tella V. (2012). Concurrent validation of the OMNI-resistance exercise scale of perceived exertion with Thera-band resistance bands. J. Strength Cond. Res..

[27] Tsuzuku S., Kajioka T., Endo H., Abbott R.D., Curb J.D., Yano K. (2007). Favorable effects of non-instrumental resistance training on fat distribution and metabolic profiles in healthy elderly people. Eur. J. Appl. Physiol..

[28] Martone A.M., Marzetti E., Calvani R., Picca A., Tosato M., Santoro L., Di Giorgio A., Nesci A., Sisto A., Santoliquido A. (2017). Exercise and Protein Intake: A Synergistic Approach against Sarcopenia. Biomed. Res. Int..

[29] Rondanelli M., Klersy C., Terracol G., Talluri J., Maugeri R., Guido D., Faliva M.A., Solerte B.S., Fioravanti M., Lukaski H. (2016). Whey protein, amino acids, and vitamin D supplementation with physical activity increases fat-free mass and strength, functionality, and quality of life and decreases inflammation in sarcopenic elderly. Am. J. Clin. Nutr..

[30] Jacob K.J., Chevalier S., Lamarche M., Morais J.A. (2019). Leucine Supplementation Does Not Alter Insulin Sensitivity in Prefrail and Frail Older Women following a Resistance Training Protocol. J. Nutr..

[31] Maltais M.L., Ladouceur J.P., Dionne I.J. (2016). The Effect of Resistance Training and Different Sources of Postexercise Protein Supplementation on Muscle Mass and Physical Capacity in Sarcopenic Elderly Men. J. Strength Cond. Res..

[32] Strandberg E., Edholm P., Ponsot E., Wåhlin-Larsson B., Hellmén E., Nilsson A., Engfeldt P., Cederholm T., Risérus U., Kadi F. (2015). Influence of combined resistance training and healthy diet on muscle mass in healthy elderly women: A randomized controlled trial. J. Appl. Physiol..

[33] Beavers K.M., Beavers D.P., Martin S.B., Marsh A.P., Lyles M.F., Lenchik L., Shapses S.A., Nicklas B.J. (2017). Change in Bone Mineral Density During Weight Loss with Resistance Versus Aerobic Exercise Training in Older Adults. J. Gerontol. Ser. A Biol. Sci. Med. Sci..

[34] Ramírez-Campillo R., Castillo A., de la Fuente C.I., Campos-Jara C., Andrade D.C., Álvarez C., Martínez C., Castro-Sepúlveda M., Pereira A., Marques M.C. (2014). High-speed resistance training is more effective than low-speed resistance training to increase functional capacity and muscle performance in older women. Exp. Gerontol..

[35] Martins W.R., Safons M.P., Bottaro M., Blasczyk J.C., Diniz L.R., Fonseca R.M., Bonini-Rocha A.C., de Oliveira R.J. (2015). Effects of short term elastic resistance training on muscle mass and strength in untrained older adults: A randomized clinical trial. BMC Geriatr..

[36] Bauer J.M., Verlaan S., Bautmans I., Brandt K., Donini L.M., Maggio M., McMurdo M.E., Mets T., Seal C., Wijers S.L. (2015). Effects of a vitamin D and leucine-enriched whey protein nutritional supplement on measures of sarcopenia in older adults, the PROVIDE study: A randomized, double-blind, placebo-controlled trial. J. Am. Med. Dir. Assoc..

[37] Addison O., Marcus R.L., Lastayo P.C., Ryan A.S. (2014). Intermuscular fat: A review of the consequences and causes. Int. J. Endocrinol..

[38] Reding K.W., Brubaker P., D’Agostino R., Kitzman D.W., Nicklas B., Langford D., Grodesky M., Hundley W.G. (2019). Increased skeletal intermuscular fat is associated with reduced exercise capacity in cancer survivors: A cross-sectional study. Cardiooncology.

[39] Hofmann M., Schober-Halper B., Oesen S., Franzke B., Tschan H., Bachl N., Strasser E.M., Quittan M., Wagner K.H., Wessner B. (2016). Effects of elastic band resistance training and nutritional supplementation on muscle quality and circulating muscle growth and degradation factors of institutionalized elderly women: The Vienna Active Ageing Study (VAAS). Eur. J. Appl. Physiol..

[40] Hanna A., Frangogiannis N.G. (2019). The Role of the TGF-β Superfamily in Myocardial Infarction. Front. Cardiovasc. Med..

